# Experimental control of transport resonances in a coherent quantum rocking ratchet

**DOI:** 10.1038/ncomms10440

**Published:** 2016-02-08

**Authors:** Christopher Grossert, Martin Leder, Sergey Denisov, Peter Hänggi, Martin Weitz

**Affiliations:** 1 Institut für Angewandte Physik der Universität Bonn, Wegelerstr. 8, 53115 Bonn, Germany; 2 Department of Applied Mathematics, Lobachevsky State University of Nizhny Novgorod, Nizhny Novgorod 603950, Russia; 3 Institut für Physik, Universität Augsburg, Universitätsstr. 1, 86159 Augsburg, Germany; 4 Sumy State University, Rimsky-Korsakov Street 2, 40007 Sumy, Ukraine; 5 Nanosystems Initiative Munich, Schellingstr. 4, D-80799 München, Germany

## Abstract

The ratchet phenomenon is a means to get directed transport without net forces. Originally conceived to rectify stochastic motion and describe operational principles of biological motors, the ratchet effect can be used to achieve controllable coherent quantum transport. This transport is an ingredient of several perspective quantum devices including atomic chips. Here we examine coherent transport of ultra-cold atoms in a rocking quantum ratchet. This is realized by loading a rubidium atomic Bose–Einstein condensate into a periodic optical potential subjected to a biharmonic temporal drive. The achieved long-time coherence allows us to resolve resonance enhancement of the atom transport induced by avoided crossings in the Floquet spectrum of the system. By tuning the strength of the temporal modulations, we observe a bifurcation of a single resonance into a doublet. Our measurements reveal the role of interactions among Floquet eigenstates for quantum ratchet transport.

## Abstract (web summary)

The ratchet effect can be used as a tool to control coherent quantum transport of ultra-cold atoms. Here, the authors demonstrate a rocking quantum ratchet with a rubidium BEC, and reveal the existence of quantum transport resonances.

## Graphical abstract

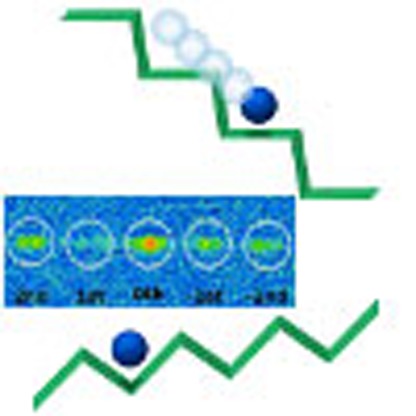

A controllable dissipationless, fully coherent quantum transport of ultra-cold atoms is a prerequisite for several applications, ranging from quantum information processing with atom chips^1^,^2^ to high-precision BEC-gravimetry^3^,^4^. There are several ways to reach this goal^5^,^6^,^7^ and the ratchet effect is one of them^8^,^9^,^10^,^11^,^12^. The essence of this effect is that a particle in a periodic potential can be set into a directed motion by using zero-mean time-periodic modulations of the potential only^13^,^14^.

There exists a variety of different ratchet devices^13^,^14^, with setup-sensitive conditions for occurrence of directed transport. Of prime importance in this context is the identification of the dynamical symmetries which prevent the appearance of the directed motion^12^. A proper choice of the system parameters, especially of the driving field, leads to the breaking of all no-go symmetries to yield an average net current.

There are two popular Hamiltonian ratchet setups for both, classical^13^,^14^ and quantum systems^12^,^15^,^16^,^17^,^18^,^19^,^20^,^21^. While flashing ratchets are characterized by multiplicative driven potentials, *U*(*x*)=*V*(*x*)*F*(*t*), rocking ratchets are realized with periodically tilted potentials, *U*(*x*)=*V*(*x*)+*F*(*t*)*x*. In the flashing mode of operation the forcing enters multiplicative, whereas it is of additive character for the rocking mode. As a consequence, the two setups belong to different dynamical symmetry groups^12^. Particularly, the rocking ratchet can be realized with a single-harmonic potential while a flashing ratchet needs a potential with at least two spatial harmonics^12^.

The symmetry analysis alone, however, fails to predict the transport direction and its average velocity. These quantities depend on the inherent mechanisms specific to the system's nature and control parameters. Physical intuition may sometimes apply, for example, a high velocity can be expected in the case of resonant driving, when the modulating frequency matches the characteristic frequency of the potential, as was verified with experiments using cold atoms in the regime of classical ratchets^22^,^23^,^24^ and, as well, with a flashing quantum ratchet realized with a Bose–Einstein condensate of rubidium atoms^11^.

An intriguing phenomenon was predicted in numerical simulations of quantum coherent ratchets^10^. Namely, the ratchet current can be substantially boosted by tuning specific Floquet states of a periodically driven potential into an avoided crossing^25^,^26^,^27^. It was also predicted that these transport resonances follow an universal bifurcation scenario upon increasing the driving strength. The scenario is dictated by generic properties of the Floquet spectra of quantum ratchets. This theoretical result provides a possibility of a more subtle (as compared with the symmetry-based scheme) control of the quantum ratchet transport. The Floquet resonances were theoretically observed with both abovementioned driving schemes^10^. However, an experimental verification requires a regime of coherent quantum transport on time scales much larger than the period of the driving.

Our objective here is the resolution of the theoretically predicted Floquet resonances in experiment, by using an a.c.-driven optical potential and an atomic Bose–Einstein condensate. In contrast to the previous experiment using a quantum flashing ratchet^11^, where a biharmonic potential building upon the dispersion of multi-photon Raman transitions was used, the rocking setup requires only a standard sinusoidal standing-wave optical potential. For alkali atoms with a *s*-electronic ground state configuration *L*=0, the absence of the second harmonic in the optical potential is beneficial because it allows for much longer coherence times as compared with those achieved with the flashing setup. Therefore, by implementing the rocking scheme, we can observe Floquet resonances in the mean velocity of ultra-cold atoms and the splitting of a single resonance into a doublet of transport resonances.

## Results

### Experimental realization

The quantum rocking ratchet is described by the time-periodic Hamiltonian^10^,^12^


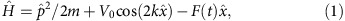


where *m* denotes the mass of the atom, *k*=2*π*/*λ* is the wave-vector of the potential, where *λ*≃783.5 nm is the wavelength of the laser beams used in the experiment, and *V*_0_ is the tunable lattice depth. A time-periodic force, *F*(*t*)=*F*(*t*+*T*), is implemented by modulating one of the two counter-propagating lattice beams with a time-dependent frequency *Ω*(*t*). In the lab frame, this field produces a moving lattice potential, *V*(*x*′,*t*)=*V*_0_ cos[2*kx*′–*f*(*t*)], 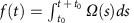, where the temporal evolution starts at the (starting) time *t*_0_∈[0,*T*]. This parameter specifies the strength of the rocking force when the modulations are switched on. In the co-moving frame, this corresponds to a stationary potential subjected to a rocking inertial force 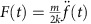, see (1). Similar to the setup in refs 22, 23, 24 we use a biharmonic frequency modulation


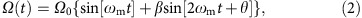


where *Ω*_0_ denotes the modulation amplitude, *ω*_m_ is the modulation frequency, and *β* and *θ* are the relative amplitude and relative phase of the second harmonic. Thus, in the co-moving frame this corresponds to the Hamiltonian in (1) with a rocking force *F*(*t*) of the form^10^


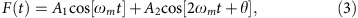


with 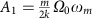 and 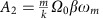.

**Figure 1:**
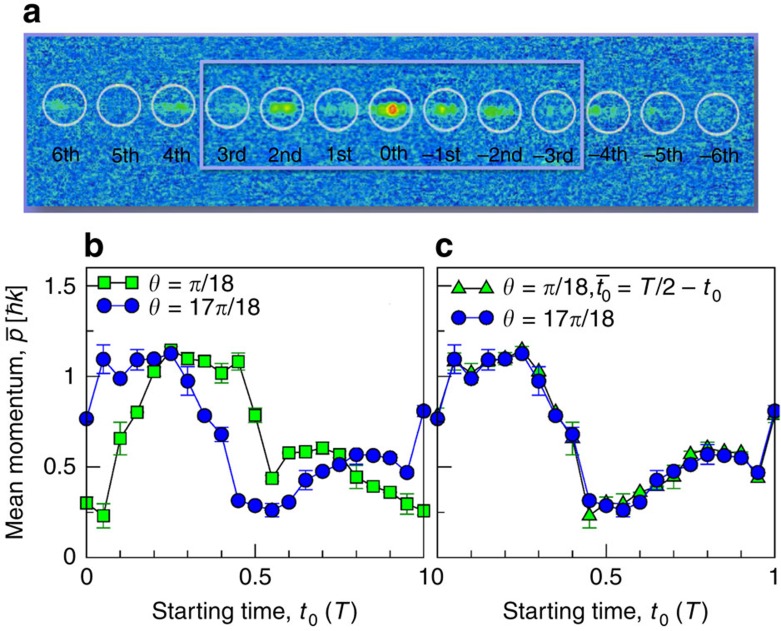
Dependence of atom transport on starting time. (**a**) Time-of-flight image recorded after 15 ms of free expansion time, showing the atomic velocity distribution after 100 modulation periods. The white circles mark the position of the visible diffraction peaks. (**b**,**c**) Mean atomic momentum as a function of the starting time *t*_0_ measured for two different values of *θ*. The shown error bars correspond to the s.d. of the mean over three measurements per data point. (**b**) Original experimental data. (**c**) The result of the transformation *t*_0_→(*T*/2–*t*_0_)mod *T* applied to the data set for *θ*=*π*/18. The measurements were performed after an interaction time of *t*=100*T*. The experimental parameters are *V*_0_=4.5*E*_r_, *Ω*_0_=241.8 kHz, *ω*_m_=24 kHz≈6.42*ω*_*r*_, *β*=12/13≈0.923. Here, the recoil energy is given by *E*_r_=(^2^*k*^2^/(2*m*) where *m* is the rubidium atomic mass, and the recoil frequency equals *ω*_r_=*E*_r_/=2π·3.74 kHz.

In our experiment, a Bose–Einstein condensate (BEC) of ^87^Rb atoms is produced first in the *m*_*F*_=0 spin projection of the *F*=1 hyperfine ground state by evaporative cooling. After that the condensate expands freely for 3 ms and converts the internal interaction energy of the dense atomic cloud into kinetic energy. From the resulting velocity distribution, a narrow slice of the momentum width Δ*p*=0.2*ℏk* is separated with a 330-μs long Raman pulse, transferring atoms into the *m*_*F*_=−1 spin projection state. The atoms are then loaded into a rocked periodic potential formed by an optical standing wave, detuned 3 nm to the red end of the rubidium *D*2-line. After the interaction with the optical potential, the atomic cloud is allowed to expand freely for 15–20 ms and then an absorption image is recorded. By using time-of-flight images, we analyse the velocity distribution of the atomic cloud, see Fig. 1. The interaction with the lattice potential during a time span [*t*_0_, *t*_0_+*t*] results in a diffraction pattern with a set of discrete peaks, separated by two photon recoils, with the *n*th order peak corresponding to a momentum of *p*_*n*_=2*ℏkn*; see Fig. 1a. The mean momentum of the atomic cloud is calculated as 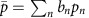, where *b*_*n*_ is the relative population of the *n*th momentum state. Due to finiteness of the contrast and sensitivity of the imaging system, we restrict the summation to *n*=−3,…,3.

### Dependence of atom current on the modulation starting time

**Figure 2:**
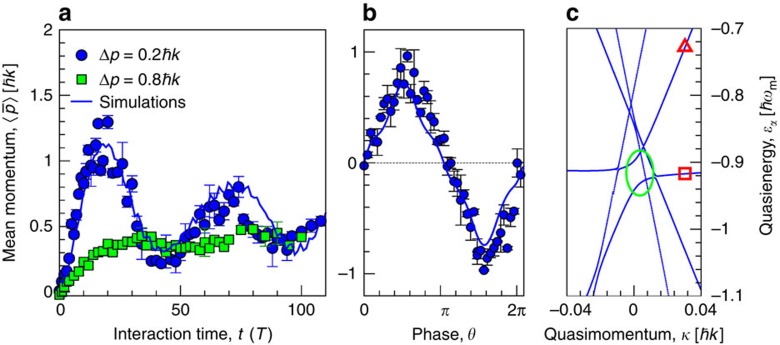
Temporal evolution of the mean atomic momentum. (**a**) Mean atomic momentum as a function of the interaction time *t* for two values of the momentum dispersion Δ*p* of the initial BEC slice. For every time instant the momentum was averaged over eight equidistant values of *t*_0_∈[0, *T*]. (**b**) Mean atomic momentum measured after an interaction time *t*=70*T* as a function of relative phase *θ* for Δ*p*=0.2ℏ*k*. The data nicely obey the symmetry property in equation (4). The thin (blue) lines in **a** and **b** correspond to the results of numerical simulations. The shown error bars correspond to the standard deviation of the averaged mean momentum. The parameters used are *V*_0_=4.45*E*_r_, *Ω*_0_=93.6 kHz, *ω*_m_=59.4 kHz≈15.9*ω*_*r*_ *β*=12/13≈0.923 and *θ*=*π*/2. (**c**) The part of the quasienergy spectrum near the centre of the first Brillouin zone (only five bands are shown). The Floquet resonance is produced by the avoiding crossing (marked by green ellipse) between two bands, the Floquet band with the minimal kinetic energy (□) and a ballistic band (▵). These corresponding two Floquet states dominantly contribute to the velocity, equation (5).

The atomic current produced by the rocking quantum ratchet, equations (1, 2, 3), can be evaluated in terms of the eigenfunctions of the operator which propagates the system over one period of the driving, *U*(*T*), 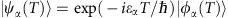. These eigenfunctions are stroboscopic snapshots of the time-periodic Floquet states {|*φ*_*α*_(*t*)}_*α*=1,2,..._, |*φ*_*α*_(*t*+*T*)〉=|*φ*_*α*_(*t*)〉 (refs 28, 29, 30). In the lab frame, the system Hamiltonian is a spatially-periodic operator and its reciprocal space is spanned by the quasienergy Bloch bands, *ɛ*_*α*_(*κ*), *κ*∈[−*π*/*L*_lat_, *π*/*L*_lat_], with *L*_lat_=*λ*/2, *α*=1,2,..., and the Floquet states are parameterized by the quasimomentum values *ℏκ*, |*φ*_*α*,*κ*_(*t*)〉(ref. 31). A non-vanishing transport is expected for *κ*=0 when 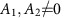 and 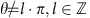. This choice of parameters results in the breaking of the sole dynamical symmetry,


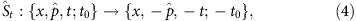


preventing the de-symmetrization of the eigenstates^8^,^9^,^10^,^11^. The average velocity of *α*-th Floquet state |*φ*_*α*,*κ*_(*t*)〉 at quasimomentum *κ* is determined by the local slope, *υ*_*α*,*κ*_=*ℏ*^−1^∂*ɛ*_*α*_(*κ*)/∂*κ* (refs 8, 9, 10). An initial wave packet can be expanded over the instantaneous Floquet basis, 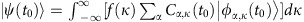. The distribution *f*(*κ*) is determined by the momentum profile of the initial wave packet, which is transformed into the profile in the *κ*-space. The velocity after an overall interaction time *t* then reads^12^


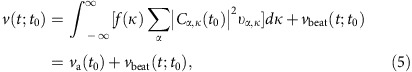


where the last term on the rhs accounts for the interference between different Floquet states. Its time average disappears in the asymptotic limit, 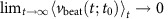, provided that either there are at least several Floquet states which overlap substantially with the initial wavefunction that is well-localized at *κ*=0, *f*(*κ*)≈*δ*(*κ*)^10^, or the initial wave packet is spread over the quasimomentum space (we discuss the corresponding mechanism in the next section). In the latter case it is enough to have two Floquet bands effectively overlapping with the initial wave packet; this latter situation is the case in our experiment; see Fig. 2c. The theoretical quantity *m*·*v*(*t*, *t*_0_) should be compared with the mean momentum of the atomic cloud, 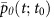, a quantity measured in the experiment and defined in the previous section.

Because of the explicit time-dependence of the Floquet states, the weights *C*_*α*,*κ*_(*t*_0_) depend on the starting time *t*_0_, so that the asymptotic velocity depends on the starting time even when the initial wave function and all other parameters are held fixed^10^,^11^. We first studied this quantum feature, namely the dependence of the atomic transport on the starting time *t*_0_∈[0, *T*], *T*=2*π*/*ω*_*m*_. Our Fig. 1b depicts the experimental results for two values of *θ*, *π*/18 (filled blue dots) and 17*π*/18 (filled green squares). In both cases we observe a strong dependence of the ratchet transport on the starting time *t*_0_. Theory predicts a particular symmetry, reading, 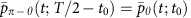. This symmetry follows from the invariance of the quantum Hamiltonian (1–3) under the transformation of *θ* and *t*_0_, combined with the double reversal {*t*,*x*}→{−*t*, −*x*} and complex conjugation. The result of this transformation applied to the experimentally measured momentum dependencies is depicted with Fig. 1c. Within experimental uncertainty, the momentum dependencies perfectly match each other. We interpret this finding as key evidence for the coherent character of the dynamics of our quantum ratchet.

### Temporal evolution of the mean atomic momentum

Figure 2a depicts the mean atomic momentum 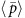, where 〈...〉 denotes the averaging of the momentum over the starting time *t*_0_, versus the number of modulation periods. The interference between the contributing Floquet states comes into play immediately after the switch-on of the modulations and induces the appearance of a non-zero current already after several periods of the modulations. Upon increasing elapsing interaction time *t*, the mean momentum exhibits several oscillations, as expected from the interference beating (note the last term on the rhs of equation (5), and saturates towards a nearly constant value. The initial wavefunction of the loaded BEC can be effectively represented as a coherent superposition of two Floquet states, see Fig. 2c. The spectral gap at the avoided crossing point (near *κ*=0), *ω*_beat_=*δɛ*/*ℏ*, *δɛ*=|*ɛ*_*α*_–*ɛ*_*β*_|, specifies the time scale of the interference beating. The theoretical model yields *ω*_beat_=0.04*ω*_m_, cf. Fig. 2c, which matches the time, *t*_max_≈20*T*, after which the first maximum appears in the dependence 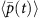 versus interaction time *t*.

The finite momentum dispersion of the BEC produces an additional, while fully coherent, damping-like effect for the time evolution of the current. Namely, contributions of Floquet eigenstates from different *κ*—bands, characterized by continuously changing quasienergies, *ɛ*_*α*_(*κ*), cf. Fig. 2c, result upon elapsing interaction time in a self-averaging of the interference term *v*_beat_ towards zero. Thus, the finite momentum width of the initial packet removes the need for the additional run-time averaging of the current in order to obtain the asymptotic velocity *v*_a_(*t*_0_) (this averaging was used in ref. 10 when calculating ratchet dynamics of the wave packet *f*(*κ*)=*δ*(*κ*)]. For a broader momentum BEC slice, this effect causes with increasingly elapsing interaction time *t* a substantial damping of the oscillations, note the filled green squares in panel Fig. 2a.

### Detection of transport resonances

We next turn to the issue of quantum transport resonances present in coherently rocking quantum ratchets, with both harmonic amplitudes 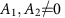. A theoretical analysis is elucidative in the limit *f*(*κ*)=*δ*(*κ*) (the analysis for the general case can be performed by using the recipe in ref. 32). When the phase 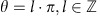, the system (1–3) obeys time-reversal symmetry so that all Floquet states become non-transporting, *υ*_*α*,*κ*=0_≡0. An asymptotic current is absent though a transient current is still possible due to the above-discussed interference effects. From the symmetry analysis of the Schrödinger equation with the Hamiltonian (1–3), it follows that the dependence of the averaged (over *t*_0_) asymptotic velocity *v*_a_(*θ*)=〈*v*_a_(*θ*;*t*_0_)〉 on θ obeys *v*_a_(*θ*)=−*v*_a_(*θ*±*π*)=−*v*_a_(−*θ*) (ref. 10). The results of experimental measurements nicely fit this theoretical prediction, see Fig. 2b.

**Figure 3:**
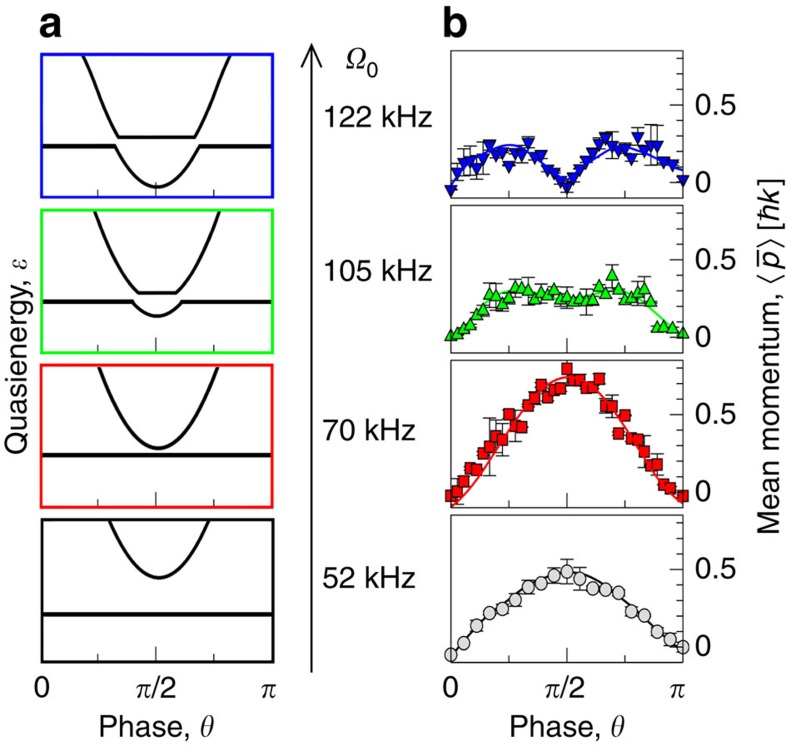
Bifurcation of a transport resonance. (**a**) A sketch of the interaction scenario between quasienergy Floquet bands (bottom to top). For low values of the modulation amplitude, the Floquet ground band (upper parabolic curve) lies far from a ballistic band (straight line). Upon increasing the modulation amplitude, the tip of the Floquet ground band approaches the ballistic band and touches the latter at the points of the maximal asymmetry *θ*=*π*/2. Because the crossing is forbidden, a further tuning of the parameter causes a bifurcation of the avoided crossing point into two avoided crossing points (for the sake of clarity, the smallness of the avoided crossings are exaggerated). The colour of the frames corresponds to the colouring used for the right panel. (**b**) Mean atomic momentum as a function of *θ* for different values of the modulation amplitude *Ω*_0_. The measurements were performed after the interaction time *t*=70*T* and averaged over eight equidistant values of *t*_0_∈[0, *T*]. The thin lines correspond to numerical results obtained for the Gaussian initial wave packet. The shown error bars correspond to the standard deviation of the averaged mean momentum. The experimental parameters are *V*_0_=3.55*E*_r_, *β*=13/7≈1.86 and *ω*_m_=16*ω*_r_.

Theoretically one may find^10^ a resonant-like increase of the average current versus *θ* when tuning the amplitude of the driving. All Floquet states are ordered with respect to their averaged kinetic energy in ascending order *α*=1,.... The Floquet state *φ*_1_(*t*) has the lowest kinetic energy and any initial wavefunction which has a lower kinetic energy overlaps with this state mainly. This state is strongly affected by the change of the potential shape and thus the corresponding dependence *ɛ*_1_(*θ*) exhibits noticeable dispersion upon the variation of *θ*, with a ‘tip', either minimum-like or maximum-like, at the point of maximal asymmetry, *θ*=±*π*/2; note the the bottom sketch in Fig. 3a. Floquet states with high kinetic energies possess large average velocities 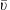. We call them ‘ballistic states'. The quasienergy dependence on *θ* of a typical ballistic state, *ɛ*_*α*=*n*_(*θ*), 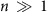, is close to a straight line because the state is only weakly affected by the variations of the potential shape.

Even when being distant on the energy scale, the two bands, *α*=1 and *α*=*n*, can be brought into an avoided crossing on the quasienergy scale, *ɛ*_*α*_∈[−*ℏω*/2, *ℏω*/2], by tuning the amplitudes of the modulating force, *A*_1_ and *A*_2_ (refs 10, 27). Due to the parabolic-like structure of the dependence *ɛ*_1_(*θ*), the two states always meet first (if they do) at the points *θ*=±*π*/2, see second (from the bottom) sketch in Fig. 3a. The eigenstates mix at these avoided crossings^33^, so that their wave functions exchange their structures. This effect leads to an increase of the average velocity of the Floquet state with minimal kinetic energy. Because the crossing is forbidden, a further increase of the modulation strength causes a bifurcation of the avoided crossing point into two avoided crossing points, with the latter moving apart upon even further increase, as it shown on third and forth (from the bottom) sketches on Fig. 3a. For an initial wavefunction which substantially overlaps with the Floquet state assuming minimal kinetic energy the ‘mixing' of the Floquet states will reveal itself through a resonance-like behaviour of the velocity dependence 〈*v*_a_〉 (ref. 10). The particular choice of the initial low-energy wave function, for example the zero-plane wave |0〉 or the ground state of the stationary potential *V*(*x*), is not essential because it does modify the results only slightly. The avoided crossing should not be sharp, however, otherwise the beating time *t*_beat_=2*πℏ*/Δ*ɛ* will be larger than the time scale of the experiment and the mixing effect cannot be detected.

Figure 3b depicts the mean momentum of the atomic cloud as a function of the phase *θ* for different values of the amplitude *Ω*_0_. For small values of *Ω*_0_, we observe an enhancement of transport at the point *θ*=*π*/2. It is attributed to a local contact of Floquet states as indicated on the two lower panels on Fig. 3a. For larger values of the amplitude *Ω*_0_, the peak splits into a doublet. This bifurcation is attributed to the splitting of a single avoided crossing point as depicted on the top panel of Fig. 3a.

**Figure 4:**
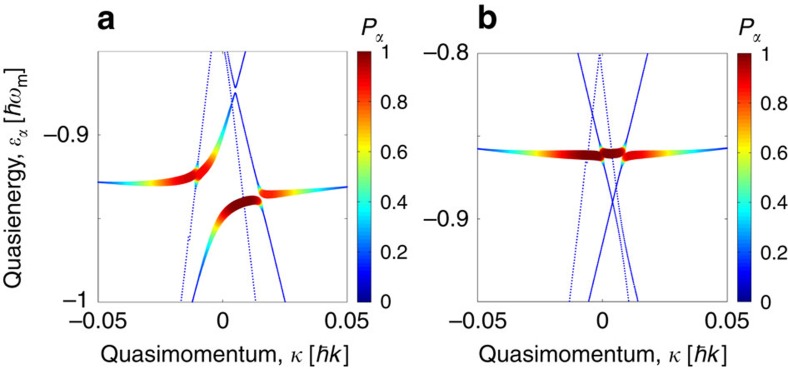
Population of the Floquet bands. The populations of the Floquet bands of the system (1–3) for two values of the modulation amplitude *Ω*_0_ (*Ω*_0_=70 kHz for **a** and *Ω*_0_=122 kHz for **b**) for a chosen relative phase *θ*=*π*/2. Width and colour of a band lines encode the relative population of the Floquet ground band and ballistic bands by the initial Gaussian wave packet (see Fig. 2c). The colour coding indicating the relative population of the *α*th state (labelled as *P*_*α*_) is given by the intensity bar. The remaining parameters used are *V*_0_=3.55*E*_r_, *β*=13/7≈1.86 and *ω*_m_=16*ω*_r_.

We also performed numerical simulations of the ratchet dynamics by using the model Hamiltonian in equations (1, 2, 3). Figure 4a,b depicts the Floquet band structure in *κ*-space at *θ*=*π*/2 obtained for the parameter set used in the experiment. The width and colour of the band indicate the relative populations (additionally averaged over starting times *t*_0_) of the bands for an initial Gaussian wave packet. The obtained numerical results confirm that for the chosen set of parameters and the chosen initial condition the system dynamics indeed is governed by two bands, a Floquet band exhibiting minimal kinetic energy (the corresponding band assumes a flat line) and a ballistic band.

For the modulation amplitude *Ω*_0_=70 kHz, Fig. 4a, there occurs an avoided crossing between the Floquet state with minimal kinetic energy and the ballistic state. This avoided crossing is responsible for the appearance of the resonant single-peak in the atomic current. On the other hand, for *Ω*_0_=122 kHz, Fig. 4b, there is almost no interaction between the ballistic state and the low-energy state. This explains the minimum in the momentum dependence at *θ*=*π*/2, cf. the top panel on Fig. 3a. The bifurcated avoided crossings are shifted from the point *θ*=*π*/2; the latter causes the formation of a double-peak pattern in the momentum dependence 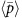 versus *θ*.

## Discussion

In conclusion, we demonstrate the control of coherent quantum transport in a rocking quantum ratchet by engineering avoided crossings between Floquet states. Rocking quantum ratchets allow for an experimentally long-lasting coherent transport regime and thus make possible the observation of specific bifurcation scenarios, such as those transport resonances. Our results give direct experimental evidence for the interaction between Floquet states of the driven system to determine the directed atomic current, enabling a fine-tuned control of transport of ultra-cold matter in the fully coherent limit.

Other problems for which coherent controllable interactions between Floquet states are beneficial include quantum systems containing a leak^34^,^35^,^36^ or photonic systems with losses^37^,^38^, where tunable external modulations can create long-lived dynamical modes. Periodically modulated optical potentials can also be put to work as tunable quantum ‘metamaterials'. This scenario also allows the sculpturing of materials with Dirac cones in the quasienergy spectrum by subtle engineering of avoided crossings between designated Floquet states. To achieve such Dirac points, the corresponding avoided crossing has to be sufficiently sharp, which means the difference between the quasienergies of the participating Floquet states, Δ*ɛ*, must assume values smaller than the characteristic time *τ* ∝ 1/*v*, where *v* is the velocity of the atomic beam moving across the optical potential. The avoided crossing in *κ*-space would then be ignored during the corresponding motion along the Floquet band^27^. Because this condition (to have a sharp avoided crossing) is opposite to what we utilized in this work (to have a broad avoided crossing, in order to resolve it on the time scale of the experiment) this latter perspective is even more appealing. The coherence time of the order 100*T* can be sufficient to meet the above condition. Possible applications of this idea include the study of Klein tunnelling^39^,^40^, or also the observation of interacting relativistic wave equations phenomena, such as a chiral confinement^41^ in a.c.-driven systems.

## Methods

### Numerical simulation

In order to reproduce the experimental measurements, we accounted for a finite width of the initial wave function and performed simulations for a Gaussian initial wave packet. If the initial packet is not too broad and well-localized within the first Brillouin zone the neglect of its tale contributions to the overall current produces a uniform rescaling of the ratchet current. This resolves the issue of the finite contrast resolution when calculating diffraction peak populations obtained in the experiments. However, it also leads to an overestimation of the current. In order to fit the measurements, we did perform numerical simulations with a Gaussian wave packet with a dispersion (being the fitting parameter used in our case) five times smaller than that used in the experiments. The region *κ*=[*ℏk*, *ℏk*] was sliced with 500 equidistant quasimomentum subspaces, assuming the initial state being in the form of the plane wave. We have also performed simulations assuming the Bloch groundsate of the undriven potential as the initial wave function (within each *κ*-slice). The obtained results only slightly differ from the presented ones. We propagate the wave functions independently and after an interaction time *t* sum the velocities by weighting them with the Gaussian distribution. Similar to the experiment, these results were averaged over eight different values of *t*_0_. The obtained dependencies are in very good agreement with the experimental data, see thin coloured lines in Figs 2a and 3b.

### Experimental details

Our experiment uses an all-optical approach to produce a quantum degenerate sample of rubidium atoms, which subsequently is loaded onto a modulated optical lattice potential to realize a rocking ratchet setup. Bose–Einstein condensation of rubidium (^87^Rb) atoms is reached by evaporative cooling of atoms in a quasistatic optical dipole trap, formed by a tightly focused horizontally oriented optical beam derived from a CO_2_-laser with optical power of 36 W operating near 10.6 μm wavelength. A spin-polarized BEC is realized by applying an additional magnetic field gradient during the final stage of the evaporation, in this case a condensate of 5 × 10^4^ atoms in the *m*_*F*_=0 spin projection of the *F*=1 hyperfine electronic ground state component^42^,^43^. A homogeneous magnetic bias of 2.9 *G* (corresponding to a Δ*ω*_*z*_≈2*π*·2 MHz splitting between adjacent Zeeman sublevels) is applied, which removes the degeneracy of magnetic sublevels.

By letting the condensate expand freely for a period of 3 ms, the atomic interaction energy is converted into kinetic energy. The measured momentum width of the condensate the atoms then reach is Δ*p*=0.8*ℏk*. We subsequently use a 330-μs long Raman pulse to cut out a narrow slice of Δ*p*=0.2*ℏk* width from the initial velocity distribution, transferring the corresponding atoms into the the *m*_*F*_=−1 spin projection. The atoms are now loaded into a modulated optical lattice potential formed by two counter-propagating optical lattice beams deriving from a high power diode laser with output power of ≈1 W detuned 3 nm to the red of the rubidium *D*2-line. Before irradiating the atomic cloud, the two optical lattice beams each pass an acousto-optic modulator and are spatially filtered with optical fibres. One of the modulators is used to in a phase-stable way modulate the relative frequency of the two lattice beams with a biharmonic function. The acousto-optic modulators are driven with two phase locked arbitrary function generators. The maximum relative frequency modulation amplitude (≈700 kHz) of the lattice beams is clearly below the Zeeman splitting between adjacent Zeeman sublevels (*ω*_*z*_/2*π*≈2 MHz), which suppresses unwanted Raman transitions between the sublevels.

After the interaction with the driven lattice, we let the atomic cloud expand freely for a 15–20-ms long period and subsequently measure the population in the *F*=1, *m*_*F*_=−1 state with an absorption imaging technique. For this, the corresponding atoms are first transferred to the *F*=2, *m*_*F*_=−1 ground state sublevel with a 34-μs long microwave *π*-pulse, and then a shadow image is recorded with a resonant laser beam tuned to the *F*=2→*F*′=3 component of the rubidium *D*2-line onto a CCD-camera, see also (ref. 43). The used time-of-flight technique allows us to analyse the velocity distribution of the atomic cloud.

## Additional information

**How to cite this article:** Grossert, C. *et al*. Experimental control of transport resonances in a coherent quantum rocking ratchet. *Nat. Commun.* 7:10440 doi: 10.1038/ncomms10440 (2016).
